# Effect of Heat Stress and Stocking Density on Growth Performance, Breast Meat Quality, and Intestinal Barrier Function in Broiler Chickens

**DOI:** 10.3390/ani9030107

**Published:** 2019-03-21

**Authors:** Doyun Goo, Jong Hyuk Kim, Geun Hyeon Park, Jomari Badillo Delos Reyes, Dong Yong Kil

**Affiliations:** Department of Animal Science and Technology, Chung-Ang University, Anseong-si, Gyeonggi-do 17546, Korea; Less1598@naver.com (D.G.); mrdgj7@naver.com (J.H.K.); pgh9210@naver.com (G.H.P.); jomdelosreyes@gmail.com (J.B.D.R.)

**Keywords:** broiler chicken, heat stress, intestinal barrier function, stocking density, tight junction-related gene expression

## Abstract

**Simple Summary:**

There is limited information on the interactive effects of heat stress (HS) and stocking density (SD) on broiler chickens. Our results indicated that both HS and high SD decreased broiler performance. HS affected intestinal barrier function by increasing intestinal permeability, but this result was not found with high SD. No interactive effects were observed between HS and SD for growth performance, meat quality, and intestinal barrier function in broiler chickens.

**Abstract:**

The present experiment was conducted to investigate the effect of heat stress (HS) and stocking density (SD) on growth performance, breast meat quality, and intestinal barrier function in broiler chickens. Experimental treatments included two different ambient temperatures (20 °C: thermoneutral conditions, or 27.8 °C: HS conditions) and two different SD (low: 9 birds/m^2^ and high: 18 birds/m^2^) in a 2 × 2 factorial arrangement. A total of 1140 21-day-old broiler chickens were allotted 1 of 4 treatments with five replicates. At the end of the experiment (35 days of age), two birds per replicate were euthanized for sample collections. The results indicated no interactions between HS and SD for all measurements. For main effects, HS decreased (*p* < 0.05) the growth performance of broiler chickens. Similarly, high SD also decreased (*p* < 0.05) body weight gain and feed intake. HS decreased (*p* < 0.01) jejunal trans-epithelial electric resistance (TER), whereas high SD did not affect TER. Neither HS nor high SD affected jejunal tight junction-related gene expressions; however, high SD reduced (*p* < 0.05) occludin expression. In conclusion, HS and high SD are key environmental factors decreasing broiler performance; however, the interactive effects of HS and high SD are not significant under the current conditions.

## 1. Introduction

In the current livestock industry, animals are raised under various stressful conditions, such as high ambient temperature, high rearing density, disease challenges, low sanitation, and improper management, which threaten the productive performance, health status, and well-being of animals. Therefore, animal researchers have made many efforts to ameliorate the stress responses of animals. However, because of limited information regarding the physiological mechanisms affecting stress responses in animals exposed to various stressors, few promising strategies have been developed.

As environmental temperature is steadily increasing worldwide, heat stress (HS) is considered one of the major challenges for the livestock industry in many countries. Among livestock animals, poultry are the most vulnerable to HS because poultry lack the ability to dissipate body heat production owing to feather covering and limited sweat glands [1]. Therefore, poultry exposed to HS frequently experience various physiological disturbances, such as systemic immune dysregulation, endocrine disorders, respiratory alkalosis, and electrolyte imbalance [2,3,4], which decrease poultry health and performance. In addition, one of the physiological challenges associated with these negative outcomes is an impaired intestinal barrier function [5,6]. Lambert [5] suggested that HS diverts blood flows from the gastrointestinal tract (GIT) to the skin, which damages the mucosal tight junction barrier in the GIT. This impaired barrier function increases intestinal permeability and, therefore, promotes the transfer of luminal endotoxins (e.g., lipopolysaccharides; LPS) into the body, which is related to an increase in local and systemic inflammatory responses.

Stocking density (SD) can also be a critical stressor in intensive poultry production because high SD is highly related to problems in the performance, health, and well-being of poultry [7,8]. The possible reasons for these problems are associated with decreased feed and water accessibility, abnormal behavior, and low air and floor quality [8,9]. Moreover, high SD can create a mild HS condition by increasing the temperature in microenvironments surrounding chickens and decreasing heat dissipation from the body [9,10]. Therefore, high SD may induce pathological responses similar to those of HS. Likewise, we previously reported that high SD decreased broiler performance and this negative effect was associated with impaired intestinal barrier function [11]. Therefore, it could be expected that the negative outcomes of high SD are exacerbated when poultry are exposed to HS. Najafi et al. [12] reported that blood stress hormones in broiler chickens were elevated when birds were raised at high SD and increased stress responses under high SD were more considerable for those birds exposed to HS. However, there is still limited information regarding the interactive effect of HS and SD in poultry. In addition, to the best of our knowledge, no experiments have been performed to study the interactive effect of HS and SD on intestinal barrier function of broiler chickens.

Therefore, the objective of the present experiment was to investigate the effect of HS and SD on growth performance, breast meat quality, and intestinal barrier function in broiler chickens. 

## 2. Materials and Methods

### 2.1. Birds, Experimental Design, and Management

The protocol for this experiment was approved by the Institutional Animal Care and Use Committee at Chung-Ang University (IACUC, approval no. 201600108). The experiment was conducted in a completely randomized design with a 2 × 2 factorial arrangement, with two different ambient temperatures and two different SD. A total of 1140 21-day-old mixed Cobb growing broiler chickens were allotted to 1 of the 4 treatments with 5 replicates. The current experiment began with 21-day-old chickens because the effect of HS and SD is typically prominent during the growing phase of broiler chickens. Birds were stocked at either 9 birds/m^2^ (low SD) or 18 birds/m^2^ (high SD). Each SD was achieved by raising different numbers of birds (i.e., 38 birds for low SD and 76 birds for high SD) per identical floor pen size (2.0 m × 2.4 m) in an environmentally controlled room [11]. In the calculation of SD, however, floor space occupied by immobile objects (0.584 m^2^ for 2 feeders and 1 bell-shaped drinker per pen) was excluded. Thus, the final floor space for each pen was 4.216 m^2^. All floor pens were covered with a 10-cm thick layer of rice hulls.

Birds from each SD were raised at either 20 °C (thermoneutral; TN conditions), or 27.8 °C (HS conditions), on average for 2 weeks (i.e., from 21 days to 35 days of age). The TN conditions were set according to the Cobb Broiler Management Guide [13]. Electric heating appliances and gas heaters were used to maintain the designed ambient temperatures. Average relative humidity was 53.0% for both temperature conditions. Before the start of the experiment, all chickens were raised under the same environmental conditions and were fed the same starter diets. During the experiment, the commercial grower diet (AME_n_, 3,130 kcal/kg; CP, 21.0%; Ca, 0.90%; available P, 0.38%; Lys, 1.33%; Met + Cys, 0.98%) was fed to all birds. Diets and water were provided ad libitum. The entire experiment was performed under 24-hour lighting conditions. Body weight gain (BWG) and feed intake (FI) were recorded at the end of the experiment. Feed efficiency (FE) was calculated as BWG divided by FI after adjusting mortality [14].

### 2.2. Sample Collection and Chemical Analysis

At the end of the experiment, 2 birds per replicate, each with a body weight (BW) close to the average BW of each replicate, were euthanized by CO_2_ asphyxiation. One bird was used for analyzing breast meat quality, serum LPS, and jejunal tight junction-related gene expression, whereas the other bird was used for analyzing intestinal permeability.

Blood samples were collected by heart puncture using a red plain tube immediately after euthanization. Blood samples were then centrifuged at 3000 × g at 4 °C for 20 minutes to separate the serum and were stored at −20 °C before LPS analysis. The serum LPS concentrations were analyzed using a Microplate Reader (Molecular Device, Sunnyvale, CA, USA) and a commercial chicken LPS ELISA Kit (My Biosource, San Diego, CA, USA). The ELISA procedure was carried out according to the protocol of the manufacturer, and the absorbance was measured at 450 nm. Mucosal samples from the jejunum of the small intestine were collected, frozen in liquid nitrogen, and stored at −85 °C until the subsequent analysis of gene expressions. For breast meat quality, the right portion of breast meat was used to analyze pH at 1 and 24 h postmortem using a pH meter (Hanna Instruments, Nusfalau, Romania), and the meat color at 24 h postmortem was measured based on the Commission Internationale de l’Eclairage (CIE) color scale for lightness (L*), redness (a*), and yellowness (b*). The left portion of the breast meat was used to measure water holding capacity (WHC) at 24 h postmortem and the thiobarbituric acid reactive substance (TBARS) value at 7 days after storage at 4 °C based on the methods described by Kim et al. [15].

The analysis of tight junction-related gene expression in the jejunal mucosa was performed with the method of Shin et al. [6]. Briefly, total RNA was extracted from the jejunal mucosa using TRIzol reagent (Invitrogen, Carlsbad, CA, USA) according to the manufacturer’s instructions. Gene expression was examined for zonula occludens-1 (*ZO-1*), occludin (*OCLN*), claudin-1 (*CLDN-1*), and junctional adhesion molecule B (*JAM-2*). A quantitative RT-PCR was performed based on the general RT-PCR method using the LightCycler 96 system (Roche, Basel, Switzerland). Gene-specific primers for target genes were designed using NCBI/Primer-BLAST and the specificity of the primers was confirmed by PCR amplification, based on the procedure demonstrated by Aznar and Alarcon [16]. The primer sequences and amplification temperatures are listed in Table 1. The relative quantification of gene-specific expression was calculated using the 2^-ΔΔCt^ method after normalization to glyceraldehyde-3-phosphate dehydrogenase (GAPDH) [17].

Intestinal permeability was measured by trans-epithelial electric resistance (TER) values in a 2-channel Ussing chamber system (U2500, Warner Instruments, Hamden, CT, USA). The detailed procedure has been reported previously [11]. In short, the mid-jejunum tissue samples (e.g., between the end of duodenal loop and Meckel’s diverticulum) collected from selected birds were placed into chilled and aerated Krebs-Henseleit buffer at pH 7.4. The jejunum was cut into sections of 3 to 5 cm, and adhering fat and mesenteries were removed. The prepared jejunal samples were then placed between the 2 halves of the Ussing chamber according to the method of Ruhnke et al. [18]. When samples were clamped in the Ussing chamber, the samples were continuously aerated with 95% O_2_ and 5% CO_2_ mixture, and completely immersed in Krebs-Henseleit buffer. The water jacket of the Ussing chamber was continuously circulated and heated to 38–39 °C. After stabilization, short circuit currents and the epithelial voltage were recorded. After 20 min of operation, TER values were calculated according to Ohm’s law and were expressed as Ω/cm^2^ [19]. 

### 2.3. Statistical Analysis

All data were analyzed by 2-way ANOVA in a completely randomized design with the MIXED procedure of SAS (SAS Institute Inc. Cary, NC, USA). The replicate was an experimental unit for all analyses. Outliers were checked using the UNIVARIATE procedure of SAS. The model included the effects of HS, SD, and their interaction. The LSMEANS procedure was used to calculate mean values. Significance for statistical test was set at *p* < 0.05.

## 3. Results

During the two-week experiment, no interactions between HS and SD were identified for any of the measurements of growth performance, breast meat quality, and intestinal barrier function. Thus, only the main effects of HS and SD were reported. 

### 3.1. Growth Performance and Breast Meat Quality

Birds raised under HS conditions had lower (*p* < 0.05) final BW, BWG, FI, and FE than those raised under TN conditions (Table 2). Breast meat quality was not affected by HS; however, breast meat from birds raised under HS conditions exhibited a greater (*p* < 0.05) 24-h postmortem pH as compared to that from birds raised under TN conditions (Table 3).

Birds raised at high SD had lower (*p* < 0.05) final BW, BWG and FI than those raised at low SD. Birds raised at high SD showed a greater WHC (*p* < 0.05) but a lower (*p* < 0.05) L* value in the breast meat than those raised at low SD. However, high SD had no effects on other meat qualities.

### 3.2. Intestinal Barrier Function

Birds raised under HS conditions had lower (*p* < 0.01) TER values than birds raised under TN conditions, indicating that HS increased intestinal permeability (Table 4). However, serum LPS concentrations, as another measure of intestinal permeability, were not affected by HS. Likewise, HS did not affect the expressions of tight junction-related genes (Table 5).

High SD had no effects on TER values and serum LPS concentrations. However, high SD decreased (*p* < 0.05) *OCLN* expression, but had no effect on *ZO-1*, *CLDN-1*, and *JAM-2* expression. 

## 4. Discussion

There were no significant interactions between HS and SD for the growth performance, breast meat quality, and intestinal barrier function of broiler chickens in the current experiment. These results were surprising because both HS and high SD are well-known stressors of broiler chickens, and were expected to have synergistic negative effects on broiler chickens. Imaeda [20] reported that broiler mortality resulting from high SD was more considerable when birds were raised under a hot season. Likewise, Najafi [12] reported that serum concentrations of stress indicators including corticosterone, ceruloplasmin, and ovotransferrin in broiler chickens were increased by high SD and the extent of increased stress responses was greater under HS conditions than under TN conditions. However, Imaeda [20] and Najafi et al. [12] observed no negative interaction between HS and SD for the growth performance of broiler chickens, which agrees with our findings that high SD decreased BWG and FI, regardless of ambient temperature, and the extent of the decreases in BWG and FI was not significantly different between HS and TN conditions. These results are difficult to explain because of limited information on the interactive effects of HS and SD on poultry. However, it is speculated that stress responses may be synergistic between HS and high SD, but it is likely insufficient to exhibit the obvious reduction in the growth performance of broiler chickens raised under the conditions of the current experiment. It should be noted that the interactive effects of HS and SD may vary with intensities and durations of HS and high SD during broiler production.

Although there were no interactions between HS and SD in this experiment, the main effects of HS and high SD were significant for some measurements of the growth performance, breast meat quality, and intestinal barrier function in broiler chickens. As expected, birds raised under HS conditions had decreased BWG and FI as compared to those raised under TN conditions, which indicated that average ambient temperature of 27.8 °C in the present experiment induced HS for growing broiler chickens. Likewise, high SD (i.e., 18 birds/m^2^) decreased BWG and FI than low SD (9 birds/m^2^), indicating that high SD in this experiment was stressful for growing broiler chickens. This result agreed with previous experiments, which reported that a SD of more than 16.0 birds/m^2^ had negative effects on the growth performance of growing broiler chickens [7,21,22]. However, the upper limits of high SD for broiler chickens may vary with breed, environment, rearing systems, experimental periods, and intensity of SD [8,12,23]. In our previous experiment [11], up to 25.3 birds/m^2^ of SD during 1 to 28 days of age had no adverse effects on the growth performance of broiler chickens raised in battery cages, whereas 18 birds/m^2^ of SD during 21 to 35 days of age in this experiment decreased the growth performance of broiler chickens raised in floor pens.

The quality of most breast meat were unaffected by HS and high SD although there were significant main effects of HS and high SD on some measurements. However, all values for breast meat quality were within the normal range of the general breast meat quality of broiler chickens. Therefore, it is likely that either HS or high SD had little negative effect on breast meat quality. However, previous experiments reported that HS produced undesirable meat quality of broiler chickens [24,25]. Thus, the reason for these inconsistent results for HS may be due to the variations in experimental factors including breed, slaughtering age, and the extent and duration of HS among experiments. However, there was little negative effect of high SD on meat quality as observed in this experiment, which is in agreement with previous experiments [11,21].

It is well documented that HS induces various physiological problems including immune dysfunctions, endocrine disturbances, respiratory alkalosis, and increased oxidative stress in poultry [2,3,4]. Moreover, HS decreases blood flow to intestinal tissues by increasing blood flow to peripheral tissues, which leads to a reduction in nutrient and oxygen supply and, therefore, impairs intestinal health and functions [5]. One of the critical functions in the GIT is to prevent the permeation of harmful substances in the lumen into the body, which is often called the intestinal barrier function [5]. Intestinal barrier function is frequently determined by intestinal permeability (i.e., gut leakage) measured with TER values for intestinal tissues and blood LPS concentrations [5,11]. Decreased TER values for intestinal tissues and increased LPS concentrations in the blood represent increased intestinal permeability. Furthermore, intestinal barrier function is highly associated with the integrity of mucosal tight junction, which is a sealing protein complex between enterocytes in the GIT [6,11]. Decreased expression of tight junction-related genes and proteins in the GIT is often correlated with decreased intestinal barrier function [6,11]. Thus, we measured jejunal TER values, serum LPS concentrations, and the expression of four important tight junction-related genes (*ZO-1*, *OCLN*, *CLDN-1*, and *JAM-2*) in the jejunal mucosa.

The observation of decreased TER values in the jejunum of broiler chickens raised under HS conditions indicated that HS increased intestinal permeability in broiler chickens. This result is in accordance with the findings of previous experiments reporting decreased intestinal TER values in broiler chickens exposed to HS [26,27]. However, high SD (i.e., 18 birds/m^2^) under the condition of the present experiment had no effect on TER values, which differs from our previous findings where increasing SD from 15.2 to 30.4 birds/m^2^ exhibited a linear reduction in jejunal TER values [11]. This inconsistent result is likely caused by the variation in rearing systems and intensity of SD among experiments. In our previous experiment, all birds were raised in battery cages under TN conditions and a reduction in TER values became more obvious when birds were raised at more than 25.3 birds/m^2^ of SD. Therefore, it is likely that 18 birds/m^2^ of SD under the current conditions was unlikely to decrease intestinal barrier function. Neither HS nor high SD influenced the serum LPS concentrations in the current experiment. It has been reported that serum LPS concentrations are negatively correlated with TER values because increased intestinal permeability accelerates the passage of luminal LPS into the body [5]. We also reported previously that serum LPS concentrations were negatively associated with TER values in broiler chickens raised in battery cages with different SD [11]. However, this relationship was not identified in the current experiment. The reason for this discrepancy is unclear; however, it may be related to less intensity of the current conditions of HS and high SD as compared to those in previous experiments [11,12,26]. In addition, variations in microbial populations among experiments may be another possible reason for inconsistent results because serum LPS originates from luminal LPS, which is a cell wall component of intestinal bacteria [5], and differences in rearing systems (battery cages vs. floor pens) and environmental temperatures can influence microbial populations [26,28]. 

*OCLN*, *CLDN-1*, and *JAM-2* are the main component proteins of tight junctions and *ZO-1* acts as a plague protein connecting between those tight junction proteins and cytoskeletons [29]. The proper expression of tight junction-related genes and proteins plays an important role in maintaining intestinal barrier function and controlling paracellular permeability [29]. Decreased expressions of tight junction-related genes and proteins in the intestinal mucosa of broiler chickens have been reported for *OCLN* and *ZO-1* proteins by HS [26] and for *ZO-1* and *JAM-2* genes by high SD [11]. However, we observed no main effects of HS and high SD on tight junction-related gene expression in the jejunal mucosa, which indicate that our experimental conditions of HS and SD cannot largely affect the integrity of mucosal tight junction. However, we found decreased TER values under HS conditions although TER values were not affected by high SD. This inconsistent finding may indicate that the expressions of tight junction-related genes and proteins are not always correlated with intestinal permeability measured by TER values. However, there is a possibility that the correlation may become obvious only when changes in the expressions of tight junction-related genes and proteins exceed a certain level. Moreover, it should also be noted that we may fail to detect significance due to the large inherent variations in tight junction-related gene expressions among individual birds because HS decreased expressions of *OCLN*, *CLDN-1*, and *JAM-2* by more than 30%, whereas high SD decreased expressions of all four genes by more than 36%. Further research is required to elucidate the relationship between intestinal permeability and the expression of tight junction-related genes and proteins.

## 5. Conclusions

No interactive effects of HS and SD are observed on the growth performance, breast meat quality, and intestinal barrier function of growing broiler chickens. In the conditions of the current experiment, both HS and high SD decrease broiler performance with little negative effect on breast meat quality. Intestinal barrier function is decreased by HS in growing broiler chickens, but the decrease in intestinal barrier function by high SD is not considerable in the present experiment. 

## Figures and Tables

**Table 1:** Primers used for quantitative RT-PCR.

Primer Name ^1^	Primer Sequence ^2^ (5′-3′)	Tm ^3^, °C	Product Size, bp	Accession Number
*GAPDH*	F: TGCTGCCCAGAACATCATCC	50.0–65.0	142	NM_204305
	R: ACGGCAGGTCAGGTCAACAA			
*ZO-1*	F: AATACCTGACTGTCTTGCAG	58.3	145	XM_015278975.1
	R: TAAAGAAGGCTTTCCCTGAC			
*OCLN*	F: TCGTGCTGTGCATCGCCATC	60.0	178	NM_205128.1
	R: CGCTGGTTCACCCCTCCGTA			
*CLDN-1*	F: CAGACTCTAGGTTTTGCCTT	58.3	149	NM_001013611.2
	R: AATCTTTCCAGTGGCGATAC			
*JAM-2*	F: GTGAATTTACAGTTCCTCCC	53.9	158	NM_001006257.1
	R: TCCTGTCTTTTCCAGTAAGG			

^1^*GAPDH*, glyceraldehyde-3-phosphate; *ZO-1*, zonula occludens-1; *OCLN*, occludin; *CLDN-1*, claudin-1; *JAM-2*, junctional adhesion molecules-B [11]. ^2^ F, forward; R, reverse. ^3^ Tm, melting temperature.

**Table 2:** Effect of heat stress and stocking density on the growth performance of growing broiler chickens from 21 to 35 days of age.

Item		Initial Body Weight (g)	Final Body Weight (g)	Body Weight Gain (g)	Feed Intake (g)	Feed Efficiency (g/kg)
Temperature ^1^	SD ^2^					
Thermoneutral	9	891	1918	1028	1902	540
	18	886	1776	890	1799	494
Heat stress	9	895	1701	806	1666	483
	18	883	1651	768	1583	486
SEM (*n* = 5)		6.1	32.7	35.3	27.9	14.0
Main effect						
Temperature						
Thermoneutral		888	1847	959	1851	517
Heat stress		889	1676	787	1624	484
SEM (*n* = 10)		4.3	23.1	25.0	19.7	9.9
SD						
9		893	1810	917	1784	511
18		884	1713	829	1691	490
SEM (*n* = 10)		4.3	23.1	25.0	19.7	9.9
*p*-Value	df					
Temperature	1	0.861	<0.001	<0.001	<0.001	0.034
SD	1	0.172	0.009	0.025	0.004	0.139
Temperature × SD	1	0.556	0.177	0.178	0.720	0.103

^1^ Average ambient temperatures were 20 °C and 27.8 °C for thermoneutral conditions and heat stress conditions, respectively. ^2^ Stocking density = number of birds per square meter (birds/m^2^).

**Table 3:** Effect of heat stress and stocking density on the breast meat quality of growing broiler chickens.

Item		Yield, %	pH, 1h	pH, 24h	WHC, %	L*	a*	b*	TBARS
Temperature ^2^	SD ^3^								
Thermoneutral	9	16.4	6.1	5.8	75.6	54.4	3.0	7.1	0.171
	18	15.0	6.1	5.8	78.4	51.4	2.5	6.3	0.201
Heat stress	9	14.7	6.3	6.0	77.5	56.2	3.4	8.4	0.195
	18	13.6	6.3	6.0	80.2	52.8	3.0	6.5	0.204
SEM (*n* = 5)		0.82	0.09	0.05	1.29	1.41	0.48	0.78	0.017
Main effect									
Temperature									
Thermoneutral		15.7	6.1	5.8	77.0	52.9	2.8	6.7	0.186
Heat stress		14.1	6.3	6.0	78.9	54.5	3.2	7.5	0.200
SEM (*n* = 10)		0.58	0.06	0.04	0.91	1.00	0.34	0.55	0.012
SD									
9		15.6	6.2	5.9	76.6	55.3	3.2	7.7	0.183
18		14.3	6.2	5.9	79.3	52.1	2.7	6.4	0.203
SEM (*n* = 10)		0.58	0.06	0.04	0.91	1.00	0.34	0.55	0.012
*p*-Value	df								
Temperature	1	0.071	0.082	0.012	0.168	0.282	0.373	0.339	0.445
SD	1	0.138	0.679	0.740	0.046	0.038	0.332	0.113	0.261
Temperature × SD	1	0.843	0.679	0.626	0.954	0.895	0.902	0.491	0.551

^1^ WHC, water holding capacity; L*, lightness; a*, redness; b*, yellowness; TBARS, thiobarbituric acid reactive substances (mg of malondiadehyde/kg). ^2^ Average ambient temperatures were 20 °C and 27.8 °C for thermoneutral conditions and heat stress conditions, respectively. ^3^ Stocking density = number of birds per square meter (birds/m^2^).

**Table 4:** Effect of heat stress and stocking density on intestinal permeability ^1^ in the jejunum of growing broiler chickens.

Item		PD, mV	Isc, μa/cm^2^	TER, Ω/cm^2^	LPS, EU/mL
Temperature ^2^	SD ^3^				
Thermoneutral	9	187	0.7	283	16.8
	18	179	0.6	303	21.8
Heat stress	9	112	0.8	145	20.2
	18	129	0.7	196	22.0
SEM (*n* = 5)		29.9	0.11	29.9	5.36
Main effect					
Temperature					
Thermoneutral		183	0.6	293	19.3
Heat		120	0.7	170	21.1
SEM (*n* = 10)		21.2	0.08	21.1	3.59
SD					
9		149	0.7	214	18.5
18		154	0.6	250	21.9
SEM (*n* = 10)		21.2	0.08	21.1	3.59
*p*-Value	df				
Temperature	1	0.052	0.482	<0.001	0.721
SD	1	0.880	0.538	0.247	0.502
Temperature × SD	1	0.677	0.627	0.613	0.751

^1^ PD, trans-epithelial voltage; Isc, short circuit current; TER, trans-epithelial electrical resistance; LPS, serum lipopolysaccharide (EU, endotoxin units/ml). ^2^ Average ambient temperatures were 20 °C and 27.8 °C for thermoneutral conditions and heat stress conditions, respectively. ^3^ Stocking density = number of birds per square meter (birds/m^2^).

**Table 5:** Effect of heat stress and stocking density on tight junction-related gene expression ^1^ in the jejunal mucosa of growing broiler chickens.

Item		*ZO-1*	*OCLN*	*CLDN-1*	*JAM-2*
Temperature ^2^	SD ^3^				
Thermoneutral	9	0.84	1.82	1.48	1.59
	18	0.95	1.14	1.30	0.98
Heat stress	9	1.52	1.32	1.49	1.29
	18	0.57	0.47	0.44	0.46
SEM (*n* = 5)		0.452	0.384	0.489	0.415
Main effect					
Temperature					
Thermoneutral		0.90	1.48	1.39	1.29
Heat		1.05	0.89	0.97	0.87
SEM (*n* = 10)		0.304	0.258	0.328	0.278
SD					
9		1.18	1.57	1.48	1.44
18		0.76	0.81	0.87	0.72
SEM (*n* = 10)		0.304	0.258	0.328	0.278
*p*-Value	df				
Temperature	1	0.728	0.120	0.364	0.297
SD	1	0.327	0.048	0.196	0.079
Temperature × SD	1	0.221	0.817	0.350	0.775

^1^*ZO-1*, zonula occludens-1; *OCLN*, occludin; *CLDN-1*, claudin-1; *JAM-2*, junctional adhesion molecules-B. ^2^ Average ambient temperatures were 20 °C and 27.8 °C for thermoneutral conditions and heat stress conditions, respectively. ^3^ Stocking density = number of birds per square meter (birds/m^2^).
